# A novel prognostic model related to oxidative stress for treatment prediction in lung adenocarcinoma

**DOI:** 10.3389/fonc.2023.1078697

**Published:** 2023-01-31

**Authors:** Haijun Peng, Xiaoqing Li, Yanchao Luan, Changjing Wang, Wei Wang

**Affiliations:** Department of Thoracic Surgery, Hebei Chest Hospital, Hebei Provincial Key Laboratory of Lung Disease, Shijiazhuang, Hebei, China

**Keywords:** lung adenocarcinoma, oxidative stress, prognostic model, machine learning, tumor microenvironment

## Abstract

**Background:**

The prognostic model based on oxidative stress for lung adenocarcinoma (LUAD) remains unclear.

**Methods:**

The information of LUAD patients were acquired from TCGA dataset. We also collected two external datasets from GEO for verification. Oxidative stress-related genes (ORGs) were extracted from Genecards. We performed machine learning algorithms, including Univariate Cox regression, Random Survival Forest, and Least Absolute Shrinkage and Selection Operator (Lasso) analyses on the ORGs to build the OS-score and OS-signature. We drew the Kaplan-Meier and time-dependent receiver operating characteristic curve (ROC) to evaluate the efficacy of the OS-signature in predicting the prognosis of LUAD. We used GISTIC 2.0 and maftool algorithms to explore Genomic mutation of OS-signature. To analyze characteristic of tumor infiltrating immune cells, ESTIMATE, TIMER2.0, MCPcounter and ssGSEA algorithms were applied, thus evaluating the immunotherapeutic strategies. Chemotherapeutics sensitivity analysis was based on pRRophetic package. Finally, PCR assays was also used to detect the expression values of related genes in the OS-signature in cell lines.

**Results:**

Ten ORGs with prognostic value and the OS-signature containing three prognostic ORGs were identified. The significantly better prognosis of LUAD patients was observed in LUAD patients. The efficiency and accuracy of OS-signature in predicting prognosis for LUAD patients was confirmed by survival ROC curves and two external validation data sets. It was clearly observed that patients with high OS-scores had lower immunomodulators levels (with a few exceptions), stromal score, immune score, ESTIMATE score and infiltrating immune cell populations. On the contrary, patients with higher OS-scores were more likely to have higher tumor purity. PCR assays showed that, MRPL44 and CYCS were significantly higher expressed in LUAD cell lines, while CAT was significantly lower expressed.

**Conclusion:**

The novel oxidative stress-related model we identified could be used for prognosis and treatment prediction in lung adenocarcinoma.

## Introduction

According to the global cancer statistics analysis in 2020, the incidence of lung cancer ranks second only to breast cancer in the world, accounting for about 18% of all cancer deaths, and being the leading cause of cancer death in the world (1). The causes of lung cancer are very complex, including history of exposure to smoking and secondhand smoke, air pollution, history of pulmonary diseases, family history of cancer, occupational exposure to silica and asbestos, poor diet, mental and psychological factors (2–4). The early symptoms of lung cancer are not obvious. Generally, there are corresponding clinical symptoms in the middle and late stage, such as chest pain, hemoptysis, etc. According to relevant studies, 75% of lung cancer patients have been diagnosed at stage III or IV, at which time they have lost the opportunity for surgery, and the treatment means are relatively limited. Conventional radiotherapy and chemotherapy have no obvious effect, and the survival and prognosis are very poor (5). The overall 5-year survival rate for patients with lung cancer is 19%, which drops to 5% if distant metastasis is present at the time of diagnosis, and approximately 57% for patients in the localized stage (6). The diagnosis and treatment of lung cancer are still the focus of current research.

According to pathological types, lung cancer can be divided into non-small cell lung cancer (NSCLC) and small cell lung cancer (SCLC). NSCLC accounts for about 85% of all cases diagnosed with lung cancer, which mainly includes lung adenocarcinoma (LUAD), lung squamous cell carcinoma (LSCC), and large cell lung cancer (LCLC) (7). LUAD is the most common pathological type of lung cancer, accounting for approximately 50-70% of surgically resected lung cancers (8) and almost 50% of all lung cancers (9). Precision medicine for disease requires accurate prognostic prediction, such as the risk of future recurrence after the initial treatment and responsiveness to different treatments (10, 11). At present, TNM staging is still the main basis for the treatment of LUAD and has been used clinically for many years as a prognostic predictor of LUAD (12). However, the reproducibility and discrimination ability of TNM staging for prognosis prediction are still not satisfied, and the prognosis is also different among LUAD patients with the same pathological type and stage. At the same time, although the emerging diagnosis and treatment technologies such as gene testing, targeted therapy and immunotherapy have been applied in the clinical diagnosis and treatment of lung cancer, the overall survival rate of lung cancer has only slightly improved compared with other malignant tumors (6, 13). Therefore, there are individual differences in LUAD, and prognosis prediction needs individual predictors.

Tumors often have oxidative stress (OXS), which is an imbalance between oxidation and anti-oxidation in the body that causes aberrant oxidative signal regulation and macromolecular oxidative damage (14). Cellular OXS is caused by ROS accumulation (15). OXS is the principal cause of cell damage, targeting intracellular macromolecules and promoting and suppressing tumor growth (14, 16–18). Tumor cell redox homeostasis control may improve tumor therapy. OXS regulates tumor cell fate in various ways that depend on tumor type and etiology. Future study will focus on controlling OXS’s anti-tumor and tumor-promoting effects. We can evaluate OXS heterogeneity in cancers and find new therapeutic targets using bioinformatics and other big data analysis methods.

With the emergence of public biomedical databases such as TCGA (The Cancer Genome Atlas) database, the use of bioinformatics to mine disease gene data has become an important direction of disease research (19). TCGA aims to focus on acquired changes of cancer genes. Up to now, a total of 33 types of cancers have been included in TCGA database (19). Clinical sample information and sequencing data (including transcriptome data, epigenetic data, genomic mutation data, etc.) of more than 20,000 patients can be accessed openly, which has become an important database for cancer research (19, 20). The gene expression data and clinical information of LUAD patients needed in this study were obtained from public databases. In this study, we obtained transcriptome and corresponding clinical data from TCGA, Genecards, and GEO databases. Firstly, Univariate Cox regression analysis was performed and oxidative stress-related genes (ORGs) affecting overall survival of LUAD were selected. Random Survival Forest and Least absolute shrinkage and selection operator (LASSO) analyses were used to screen and construct the OS-signature. We carried out efficacy evaluation for the OS-signature of LUAD using Kaplan-Meier and receiver operating characteristic (ROC) curves and the LUAD-cohort from GEO was used to validate the OS-signature. In addition, we evaluated the somatic mutation, genomic mutation, immunological characteristics, and sensitivity to chemotherapy for OS-signature. Finally, Quantitative Real-time PCR assays were used to detect the expression of the three genes establishing the OS-signature in LUAD cell lines.

## Materials and methods

### Collection and preprocessing the data of lung adenocarcinoma

The Cancer Genome Atlas (TCGA) is a major government-funded research initiative funded by the National Cancer Institute (NCI) and the National Human Genome Research Institute (NHGRI) (21). Transcripts and clinical information of lung adenocarcinoma (LUAD) were extracted from TCGA (https://xenabrowser.OS/) (19, 22). We excluded LUAD patients without information of OS (Overall Survival), thus obtaining the clinical information and expression profiles of 502 LUAD patients. The data form of fragments per kilobase of transcript per million fragments mapped (FPKM) was transformed into transcripts per kilobase million (TPM) (22). We also used GEO data, including GSE37745 and GSE31210, generated from Affymetrix Human Genome U133 Plus 2.0 chip based on GPL570 platform as external validation groups (23). Genecards (https://www.genecards.org) is a comprehensive searchable gene database, where we can obtain information about almost all known human genes (24, 25). In order to obtain genes related to oxidative stress (oxidative stress related genes, ORGs), we set the screening threshold as relevance score>20 (26).

### Establishment of the OS-signature for LUAD

After collection and preprocessing the data of LUAD, the Univariate Cox regression analysis was performed on the ORGs collected to identify ORGs with prognostic value (prognostic ORGs, P<0.05) (27). We used randomForestSRC package in R to execute Random Survival Forest (RSF) analysis, thus filtrating prognostic ORGs with greater value (variable importance>0.25) (28). Least absolute shrinkage and selection operator (LASSO) analysis is a compression estimation method for linear model (29). The regression coefficients can be compressed by minimizing the sum of residual squares under the constraint that the sum of absolute values of various coefficients is less than a constant, thus getting a sparse model (29). This model can effectively select variables for high dimensional and collinearity data (30). The Cox regression model for LASSO analysis provided by glmnet package in R software (31) was used to calculate the OS-scores and construct the prognostic OS-signature for LUAD.

### Efficacy evaluation for the OS-signature of LUAD

The survminer package in R software was used to select the best separated value (cutoff value) of gene expression or OS-scores. The survival curves (Kaplan-Meier curves) of the high- or low-risk groups were drawn, and the survival differences between the two groups were compared (32). The receiver operating characteristic curve (ROC) is also known as the sensitivity curve (33). The research method is to analyze the Area Under the ROC Curve (AUC) of the research objects to judge the recognition ability of different diagnostic test objects for diseases. The timeROC package of R software was used to draw time-dependent (1-, 3-, and 5-year) ROC curves to evaluate the diagnostic efficacy and predictive effect of OS-signature for LUAD.

### Genomic mutation analysis for OS-signature in LUAD

Somatic mutation and copy number variation (CNV) data of LUAD patients were downloaded from cBioPortal (http://www.cbioportal.org/datasets) (34) and FireBrowse (http://firebrowse.org/) (35) respectively. To determine the mutational burden in LUAD patients, the total number of non-synonymous mutations in LUAD was calculated. Somatic alterations of driver genes in LUAD were evaluated by OS-signature grouping. The R software package maftool was used to identify the driver genes of LUAD and analyze the top 20 driver genes with the highest frequency of change. We assessed genomic characteristics by Genomic Identification of Significant Targets in Cancer 2.0 (GISTIC 2.0, https://gatk.broadinstitute.org) analysis (36).

### Characteristic analysis of tumor infiltrating immune cells

According to the transcriptome expression data from TCGA-LUAD cohort, the single sample gene set enrichment analysis (ssGSEA) algorithm in R package GSVA (Gene Set Variation Analysis) was used to rank the genes contained in the sample according to their expression level from high to low, and the rank of all genes was obtained (37). Each type of immune cell is characterized by a separate subset of genes. In this study, 783 genes were used to characterize 28 common immune infiltrating cell types. According to the background gene sets generated by each sample and arranged according to the expression situation, the enrichment scores of all samples for 28 types of immune infiltrating cells in each subset could be obtained by systematic calculation (38, 39). The advantages of this method are that it uses gene sets instead of single genes to annotate immune cell subsets and combined with multiple validation methods to improve the annotation accuracy of enrichment scores. The ESTIMATE ((The Estimation of Stromal and Immune cells in Malignant Tumor tissues using Expression) method was used to evaluate the ESTIMATE score, immune score, and stromal score of each LUAD patient (40). Besides, we assessed the levels of six kinds immune infiltrating cells (B cell, T cell CD4, T cell CD8, Neutrophil, Macrophage, and DC) *via* Tumor Immune Estimation Resource 2.0 (TIMER 2.0; http://timer.cistrome.org/) (41). We also used the MCPcounter algorithm to estimate the relative proportions of ten immune cells (T cells, CD8 T cells, Cytotoxic lymphocytes, B lineage, NK cells, Monocytic lineage, Myeloid dendritic cells, Neutrophils, Endothelial cells, and Fibroblasts) in LUAD (42). We extracted seven kinds of immunomodulators (Antigen presentation, Cell adhesion, Co-inhibitor, Co-stimulator, Ligand, Other, and Receptor) from previous study to explore the association between OS-scores and immune processes (43).

### Chemotherapeutics sensitivity analysis for OS-signature in LUAD

The Genomics of Drug Sensitivity in Cancer (GDSC) database was used to screen the wide range of chemotherapeutics for LUAD (44). The prediction model was constructed based on Ridge’s regression between drug sensitivity and expression profile of cell lines using pRRophetic algorithm (45, 46). Subsequently, we calculated the IC50 value of corresponding chemotherapeutics for each LUAD patients.

### Quantitative real-time PCR assays detecting gene expression in cell lines

The human normal lung epithelial cells named BEAS-2B was supplied by Beyotime Biotechnology (Hangzhou, China). The LUAD cell lines, including A-549 and NCI-H1299, were purchased from National Collection of Authenticated Cell Cultures (Shanghai, China). BEAS-2B and NCI-H1299 were cultured in 90% RPMI (Roswell Park Memorial Institute)-1640 with 10% FBS (fetal bovine serum). A-549 was cultured in 89% F-12K + 10% FBS + 1% Glutamax. We extracted the total RNA of the cell lines by RNAsimple Total RNA Kit (Tiangen, China). Whereafter, to acquire cDNA, we reverse transcribed the cell RNA that we have obtained applying PrimeScript RT reagent Kit (Takara, Otsu, Japan). Finally, based on the premixed system of 2 μL cDNA with SYBR Premix Ex Taq (Takara, Otsu, Japan) and primers, we detected the expression values of related genes in cell lines by Applied Biosystems StepOne Plus Real-Time PCR system (Life Technologies, Grand Island, NY, USA). The primers of the target gene were supplied by Sangon Biotech (Shanghai, China). The sequences of the primers used were listed in **Table 1**.

**Table 1:** The primer sequences in PCR analysis.

Symbol	Sequences (5’-3’)
MRPL44-F	TTGAAGACGAGTACCCAGACA
MRPL44-R	GGGCTCCAATAACTGCAAAGAA
CYCS-F	CTTTGGGCGGAAGACAGGTC
CYCS-R	TTATTGGCGGCTGTGTAAGAG
CAT-F	TGGGATCTCGTTGGAAATAACAC
CAT-R	TCAGGACGTAGGCTCCAGAAG
GAPDH-F	GGAGCGAGATCCCTCCAAAAT
GAPDH-R	GGCTGTTGTCATACTTCTCATGG

## Results

### Establishment of OS-signature for patients with LUAD

**Figure 1:**
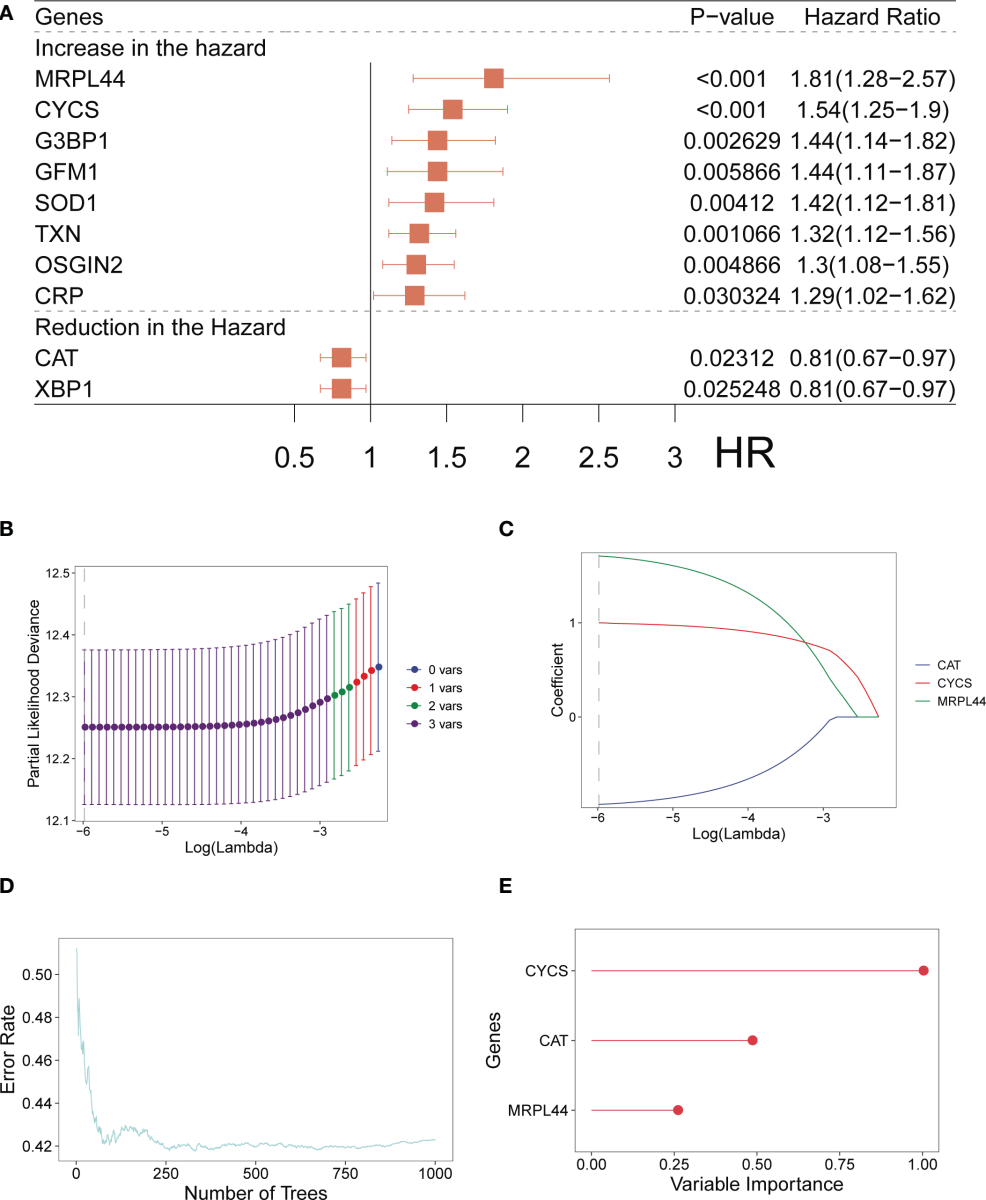
Establishment of OS-signature for patients with LUAD. **(A)** Forest plot for Univariate Cox regression analysis identifying ten oxidative stress related genes (MRPL44, CYCS, G3BP1, GEM1, SOD1, TXN, OSGIN2, CRP, CAT, and XBP1). **(B, C)** Lambda selection diagram for Least Absolute Shrinkage and Selection Operator (Lasso) analysis identifying three oxidative stress related genes (CAT, CYCS, and MRPL44) in the OS-signature. **(D)** The distribution of error rates in Random Survival Forest model. **(E)** The distribution of the variable relative importance of 12 TRP-related genes (variable importance>0.25).

For LUAD, we carried out the Univariate Cox regression analysis on a total of 80 OXRGs matched (relevance score>20). We identified a total of ten OXRGs with prognostic value (**Figure 1A**), including eight prognostic genes with HR>1 (MRPL44, CYCS, G3BP1, GFM1, SOD1, TXN, OSGIN2, and CRP) and two prognostic genes with HR<1 (CAT and XBP1). Hence, we observed eight malignant factors and two protective factors for patients with LUAD (**Figure 1A**). Whereafter, we conducted Lasso (**Figures 1–C**) and RSF (**Figures 1D–E**) analyses on the ten prognostic ORGs gained. The OS-signature ended up containing three genes: CAT, CYCS, and MRPL44 (**Figures 1B–E**). The three prognostic ORGs selected above were weighted by the regression coefficients of Lasso regression model, and finally the calculation formula of OS-signature for prognosis assessment of LUAD was obtained: OS-score =1.0002*CYCS - 0.9272*CAT + 1.7096*MRPL44. **Figures 1B, C** displayed the lambda selection diagram of the three genes in the OS-signature. The distribution of error rates generated by RSF analysis was shown in **Figures 1D, E**.

### Evaluating the efficacy of OS-signature for LUAD

**Figure 2:**
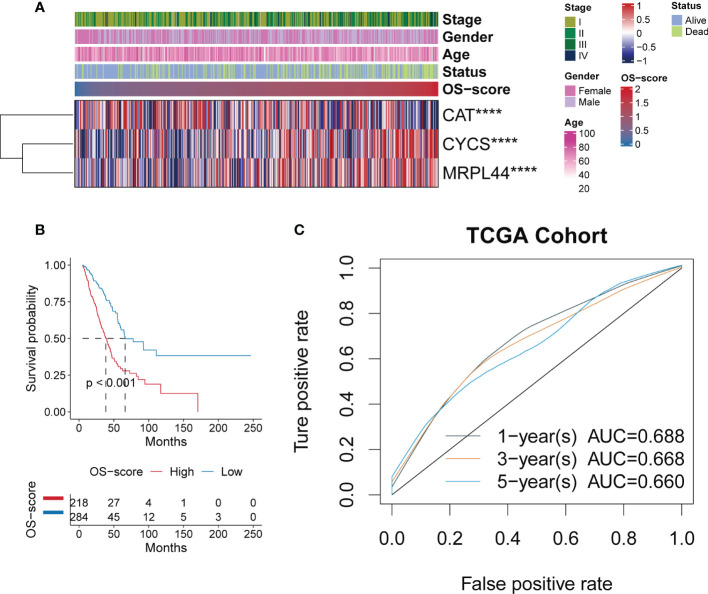
Evaluating the efficacy of OS-signature in TCGA for LUAD. **(A)** The heatmap displaying the distribution of the three oxidative stress related genes (CAT, CYCS, and MRPL44) in the OS-signature, clinicopathological characteristics (Stage, Gender, Age, Survival Status), and OS-score. Red represents high gene expression and blue represents low gene expression. **(B)** Kaplan-Meier curves displaying the correlation between the OS-score and LUAD patients. The blue curve represents the patients with lower OS-score, and the red curve represents patients with higher OS-score. **(C)** The 1-year (0.688), 3-year (0.668), 5-year (0.660) survival ROC curves predicted by the OS-signature. Different colored curves represent different years. ****p<0.0001.

**Figure 3:**
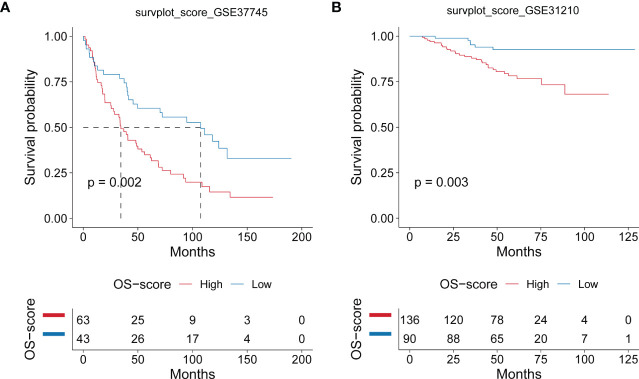
Evaluating the efficacy of OS-signature in GEO for LUAD. **(A, B)** Kaplan-Meier curves displaying the correlation between the OS-score and LUAD patients in GSE37745 **(A)** and GSE31210 **(B)**. The blue curve represents the patients with lower OS-score, and the red curve represents patients with higher OS-score.

After establishing the OS-signature based on three prognostic ORGs (CAT, CYCS, and MRPL44) for LUAD, we computed the OS-score for each LUAD patient based on the LASSO coefficients and expression value for each ORG. We compared the OS-score of LUAD patients in TCGA database among clinical features (Stage, Gender, Age and Survival Status) and the expression values of the three ORGs included in the OS-signature, which was shown in the heatmap (**Figure 2A**). Overall, patients with high OS-scores were more likely to have high expression of MRPL44 and CYCS, whereas patients with high OS-scores were strongly associated with low expression of CAT (**Figure 2A**). Kaplan-Meier analysis was used to analyze the survival and prognosis of LUAD patients in TCGA. As shown in the **Figure 2B**, patients with low OS-score had a better prognosis, while patients with high OS-score had a worse prognosis (**Figure 2B**). The AUCs of 1-year (AUC=0.688), 3-year (AUC=0.668), and 5-year (AUC=0.660) survival ROC curves predicted by the OS-signature were all larger than 0.66, suggesting the efficiency of OS-signature in predicting prognosis for LUAD to a certain extent (**Figure 2C**). To further verify the conclusion, two independent external datasets (GSE37745 and GSE31210) were included in our study, and the significantly better clinical outcomes of LUAD patients with lower OS-scores were observed (**Figures 3A, B**). Therefore, OS-signature may serve as a malignancy factor for LUAD.

### Genomic mutation analysis for OS-signature in LUAD

**Figure 4:**
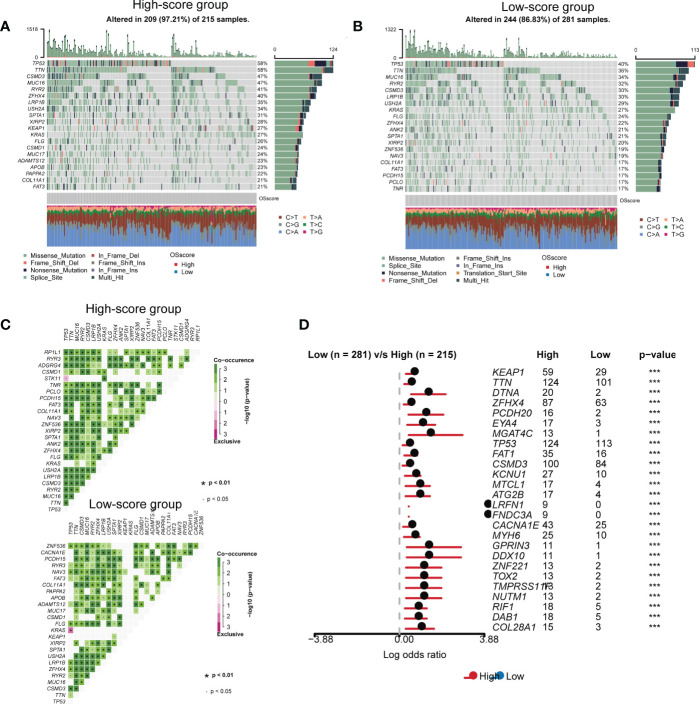
The somatic mutation features of the established OS-signature for LUAD. **(A, B)** The waterfall plot of somatic mutation features established with high **(A)** and low **(B)** OS-score. **(C)** We performed Pair-wise Fisher’s Exact test to detect mutually exclusive or co-occurrence events. **(D)** Forest plot for mutation differences between cohorts.

We carried out genomic mutation analysis for OS-signature in LUAD. From the waterfall diagram (**Figures 4A, B**), we could find that TP53, TTN, CSMD3, MUC16, RYR2, ZFHX4, LRP1B, USH2A, SPTA1, XIRP2, KEAP1, KRAS, FLG, CSMD1, MUC17, ADAMTS12, APOB, PAPPA2, COL11A1, and FAT3 were the top 20 genes with the highest mutation rate in LUAD patients with high OS-scores (**Figure 4A**). TP53, TTN, MUC16, RYR2, CSMD3, LRP1B, USH2A, KRAS, FLG, ZFHX4, ANK2, SPTA1, XIRP2, ZNF536, NAV3, COL11A1, FAT3, PCDH15, PCLO, and TNR were the top 20 genes with the highest mutation rate in LUAD patients with low OS-scores (**Figure 4B**). Thus, the mutation rates of TP53, TTN, MUC16, RYR2, ZFHX4, LRP1B, USH2A, SPTA1, XIRP2, KRAS, FLG, COL11A1, and FAT3 in the two subgroups were both relatively high. We performed Pair-wise Fisher’s Exact test to detect mutually exclusive or co-occurrence events (**Figures 4C, D**). We also Draw forest plot for mutation differences between cohorts.

**Figure 5:**
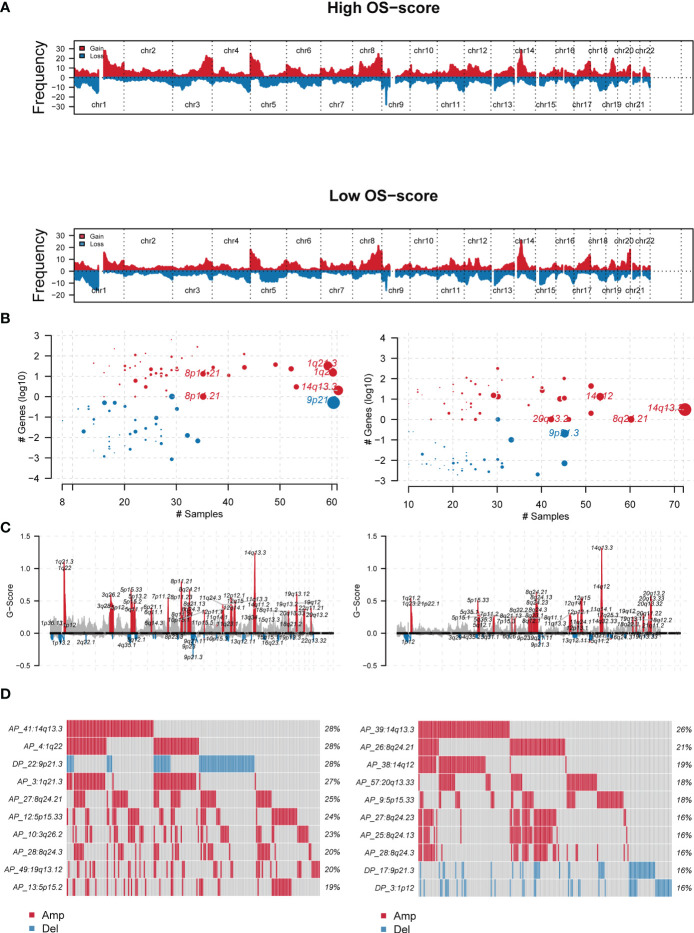
The genomic mutation analysis of the established OS-signature for LUAD. **(A)** Genomic characterization landscape of groups with high OS-scores or low OS-scores. **(B)** Plots significantly altered cytobands as a function of number samples in which it is altered and number genes it contains. Size of each bubble is according to -log10 transformed q values. **(C)** A genomic plot with segments highlighting signififcant Amplifications and Deletion regions. **(D)** The detailed amplificated or deleted CNV onco-plots of groups with high OS-scores or low OS-scores. #p<0.05.

Genomic characterization landscapes of LUAD patients with high OS-scores or patients with low OS-scores were analyzed by GISTIC algorithm and shown in **Figure 5A**. **Figure 5B** showed the plots significantly altered cytobands as a function of number samples in which it is altered and number genes it contains. **Figure 5C** showed a genomic plot with segments highlighting significant Amplifications and Deletion regions. Further, we drew the detailed amplificated or deleted CNV onco-plots of high OS-score and low OS-score subgroups (**Figure 5D**).

### Characteristic analysis of tumor infiltrating immune cells

**Figure 6:**
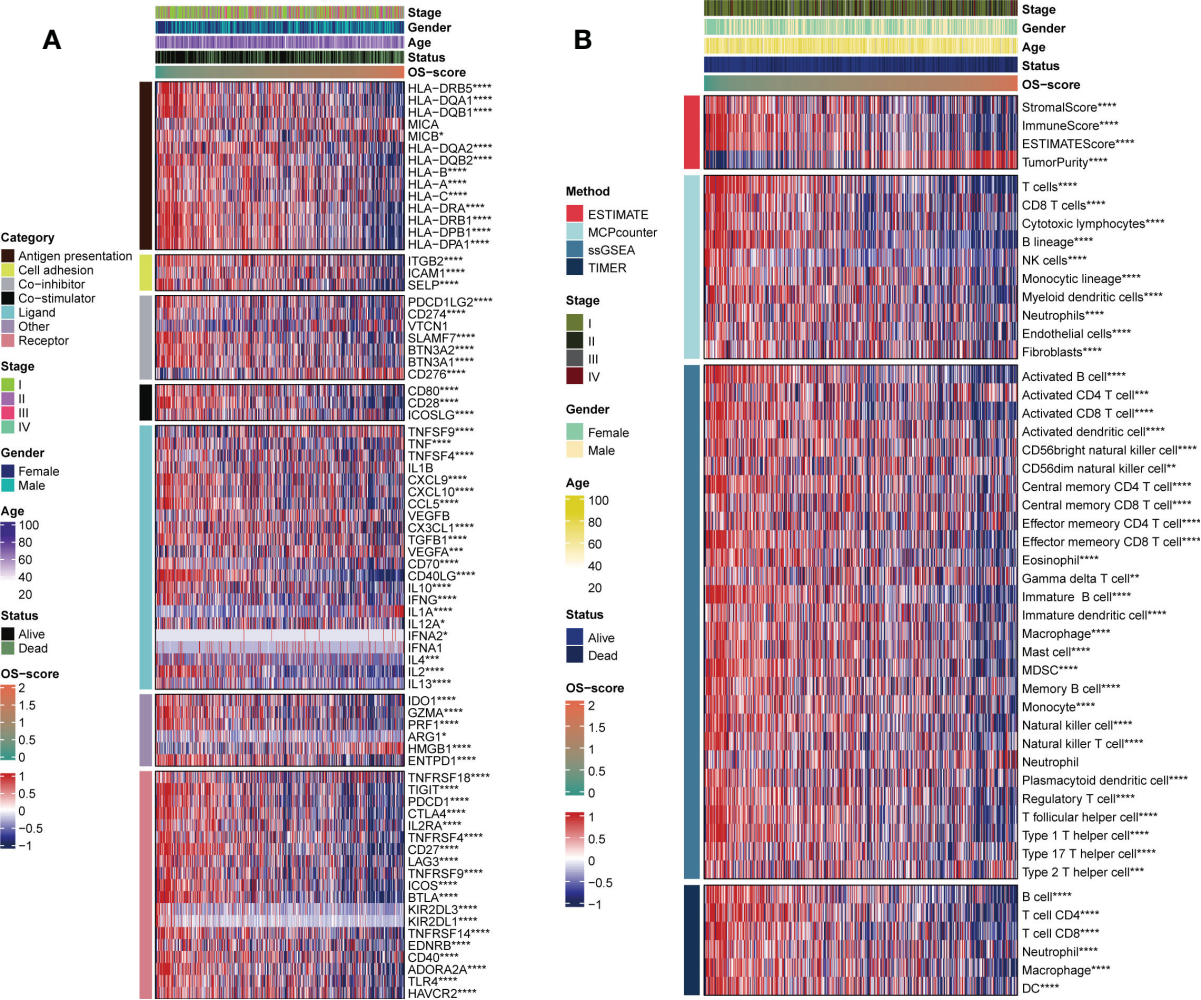
Evaluation of immunological characteristics for OS-signature. **(A)** Correlation of OS-scores with seven immunomodulators in LUAD. Red represents high enrichment score, and blue represents low enrichment score. **(B)** Heatmap displaying the abundance of infiltrating immune cell populations with different OS-scores. *p<0.05, ***p<0.001, ****p<0.0001.

Since immunomodulators (IMs) play a critical role in tumor immunotherapy, we assessed the correlation between the IMs levels (Antigen presentation, Cell adhesion, Co-inhibitor, Co-stimulator, Ligand, Other, and Receptor). It was clearly observed that patients with high OS-scores had lower IMs levels, with a few exceptions, such as CD276, TNFSF9, and HMGB1 (**Figure 6A**). From a general view, the level of stromal score, immune score, ESTIMATE score and infiltrating immune cell populations decreased as the OS-scores increased (**Figure 6B**). It was worth mentioning that patients with higher OS-scores were more likely to have higher tumor purity (**Figure 6B**).

### Chemotherapeutics sensitivity analysis for OS-signature in LUAD

**Figure 7:**
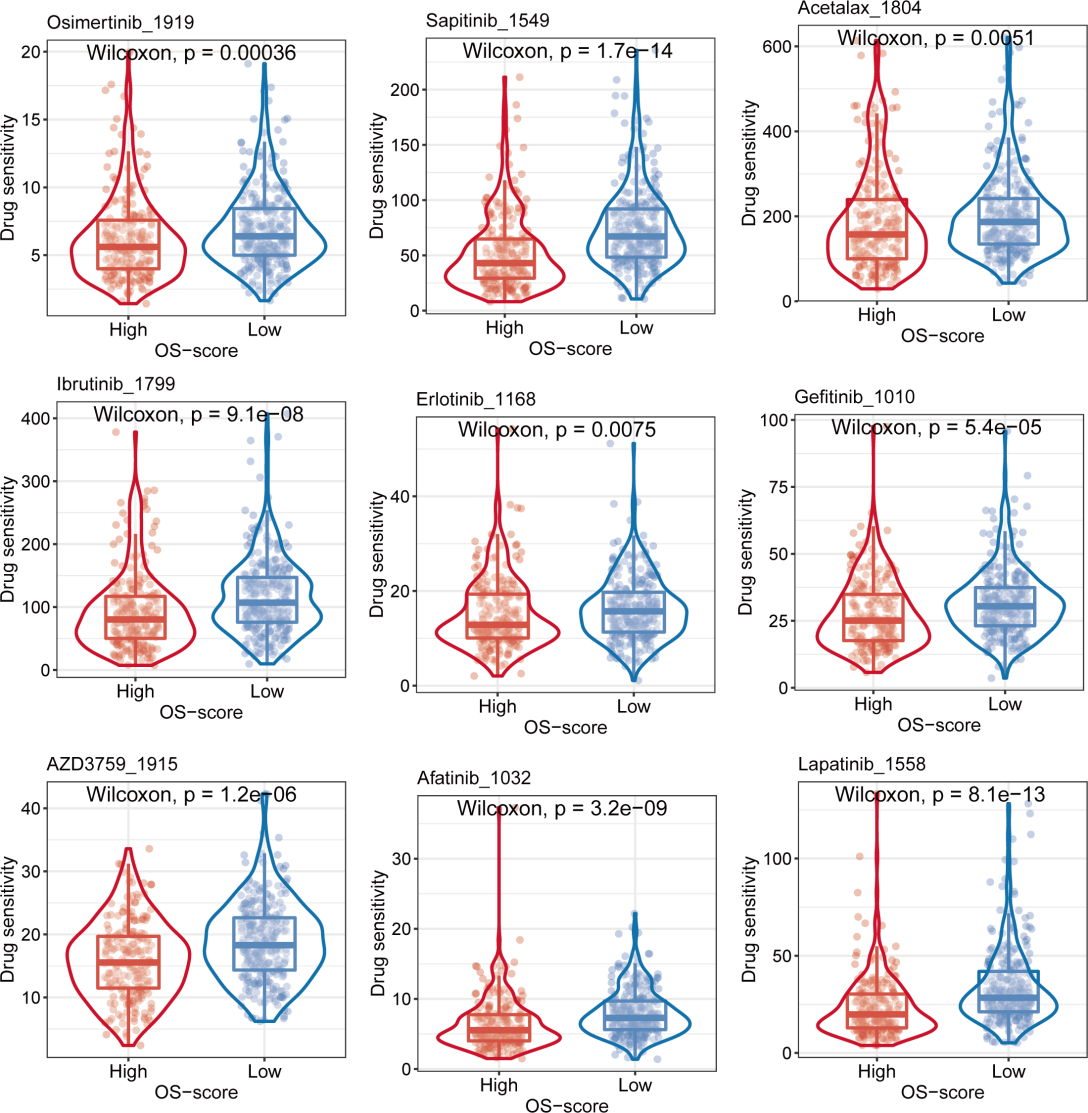
Evaluation of sensitivity to chemotherapy for OS-signature.

In order to find more effective chemotherapeutics drugs for LUAD patients with high OS-scores, we evaluated the differences in chemotherapeutics sensitivity between subgroups with high OS-score or low OS-score as described in the MATERIALS AND METHODS. The IC50 levels of nine chemotherapy drugs (Osimertinib_1919, Sapitinib_1549, Acetalax_1804, Ibrutinib_1799, Erlotinib_1168, Gefitinib_1010, AZD3759_1915, Afatinib_1032, and Lapatinib_1558) were compared between subgroups with high OS-score or low OS-score. We found that the IC50 values of the nine chemotherapy drugs were lower in LUAD patients with high OS-scores than that of LUAD patients with low OS-scores, suggesting LUAD patients with high OS-scores may be more sensitive to these nine chemotherapeutics drugs (**Figure 7**).

### Quantitative real-time PCR

We selected the three genes in the OS-signature to detect their expression in cell lines.

**Figure 8:**
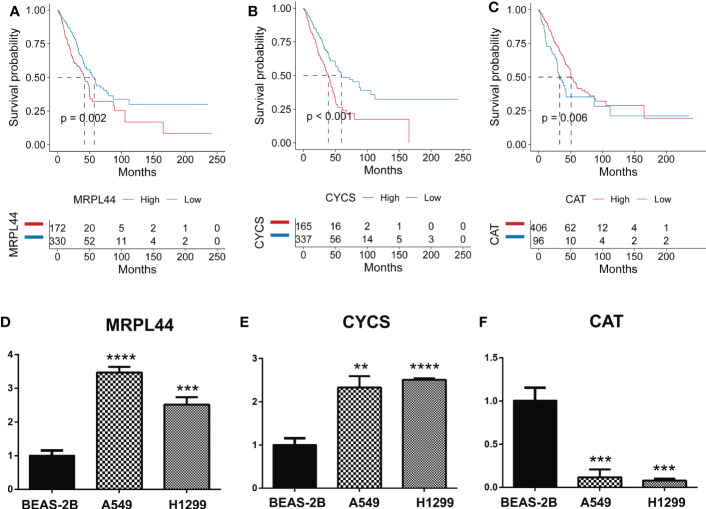
Quantitative Real-time PCR. **(A–C)** Kaplan-Meier curves displaying the correlation between the expression of the signature genes, including MRPL44 **(A)**, CYCS **(B)**, and CAT **(C)**, and the survival status of LUAD patients. The blue curve represents the patients with lower gene expression, and the red curve represents patients with higher gene expression. **(D–F)** Quantitative Real-time PCR assays using cell lines for MRPL44 **(D)**, CYCS **(E)**, and CAT **(F)**. **p<0.01, ***p<0.001, ****p<0.0001.

As could be seen from the survival curves, the higher the expression of MRPL44 (**Figure 8A**) and CYCS (**Figure 8B**), the worse the prognosis, while the opposite was true for CAT (**Figure 8C**). Compared with control cell lines (BEAS-2B), MRPL44 (**Figure 8D**) and CYCS (**Figure 8E**) were significantly higher expressed in cancer cell lines (A549 and H1299), while CAT (**Figure 8F**) was significantly lower expressed.

## Discussion

Lung cancer is a malignant tumor originating from the bronchial epithelium. According to histopathological classification, lung cancer is divided into non-small cell lung cancer (NSCLC) and small cell lung cancer (SCLC). NSCLC is the main pathological type of lung cancer, and lung adenocarcinoma (LUAD) accounts for the vast majority of NSCLC. Lung cancer ranks second only to breast cancer in incidence and is the most important cause of cancer-related deaths. Late diagnosis, poor sensitivity to chemoradiotherapy, acquired resistance to targeted therapy and other related factors can lead to poor prognosis of patients with lung cancer (47, 48). At present, histopathological diagnosis and tumor staging system are still the main basis for predicting the prognosis and survival of lung cancer patients. However, traditional methods cannot accurately assess the prognosis of patients with LUAD. In addition, Computed Tomography (CT) and serum tumor markers such as carcinoembryonic antigen (CEA) are often used to determine the prognosis of lung cancer. However, traditional methods are limited by cumulative radiation damage, low sensitivity and specificity (49, 50). Therefore, clinicians need an accurate prognostic prediction model to help optimize the treatment strategy of LUAD patients. Bioinformatics is one of the emerging fields of biological research. It uses mathematics, statistics and computer technology to process and analyze biological data. In our study, extracting data from public database, we identified eight prognostic genes with HR>1 (MRPL44, CYCS, G3BP1, GFM1, SOD1, TXN, OSGIN2, and CRP) and two prognostic genes with HR<1 (CAT and XBP1). And the OS-signature could be used for prognosis and treatment prediction in LUAD.

Because of the functional correlation between genes in a cell, diseases are rarely the result of abnormalities in a single gene, but rather result from abnormalities in a complex intracellular gene network (51–53). Like most diseases, the occurrence and development of LUAD is a complex process involving multiple genes and multiple pathogenic mechanisms, involving the activation of proto-oncogenes and the inactivation or mutation of tumor suppressor genes (54, 55). Therefore, the application of network for gene interaction in LAC research can simplify and visualize complex and high-throughput data. Compared with the focus on local gene function in single gene and single molecule biological research methods, network analysis focuses more on the integrity and systematization of biological processes (51). In the OS-signature, there were three ORGs (CAT, CYCS, and MRPL44), forming a network to predict the prognosis of LUAD. It is more reliable to explore the occurrence and development of LUAD from the perspective of multiple genes.

For early and mid-stage NSCLC that cannot be completely resected by surgery, and for some locally evolved or metastatic NSCLC that is advanced or advanced (stage IIIA-IV), comprehensive systemic and local combination therapy can be used, including surgical resection, chemotherapy, radiotherapy, targeted therapy and immunotherapy. At present, the 3rd generation chemotherapy drugs, including Docetaxel, Vinorelbine, Gemcitabine and Paclitaxel, have been widely used in clinical practice, combining platinum drugs to develop personalized treatment plans for patients. Radiotherapy is an effective means of local treatment of lung cancer, which plays a positive role in slowing down the clinical symptoms, prolonging the survival time and improving the quality of life of patients with advanced lung cancer. These treatment methods have been widely studied and applied at home and abroad. Genomic studies have shown that adenocarcinoma and squamous cell carcinoma have significantly different gene mutation types, and tyrosine kinase inhibitor (TKI) can be used to inhibit the catalytic phosphorylation of the corresponding kinases in the treatment of NSCLC patients with significant clinical benefits. Genomic studies have shown that adenocarcinoma and squamous cell carcinoma have significantly different gene mutation types, and tyrosine kinase inhibitor (TKI) can be used to inhibit the catalytic phosphorylation of the corresponding kinases in the treatment of NSCLC patients with significant clinical benefits (56, 57). A variety of effective and well-tolerated TKIs targets, including EGFR, ALK, ROSI, HER2, etc., have emerged continuously, and promoted significant progress in cancer treatment. For example, EGFR driver gene mutations have a high incidence in various subtypes of NSCLC. The most common EGFR mutations include exon19 deletion (delE746-750, etc.) or exon 21 arginine substitution leucine (L858R) mutation. EGFR inhibitors such as Gefitinib, Erlotinib, Afatinib, or Osimertinib play an important role in the treatment of NSCLC patients (58). However, some studies have shown that the proportion of NSCLC patients carrying EGFR mutations is about 30-40%, and there are still a large number of patients who cannot benefit directly from targeted therapy (59). With the development of Crizotinib and next-generation ALK-TKIs, considerable progress has been made in the treatment of patients with ALK recombinant NSCLC (60). Crizotinib, a first-generation ALK inhibitor originally approved for patients with ALK-positive NSCLC, was found to have a median progression-free survival of 8-10 months in treated patients (61). Subsequent randomized controlled trials compared Crizotinib with chemotherapy in patients undergoing treatment with a significant improvement in progression-free survival. Subsequently, second-generation ALK inhibitors Ceritinib, Alectinib and Brigatinib were developed to overcome Crizotinib resistance in patients (62). So far, other treatments, including third-generation ALK inhibitors Lorlatinib, Entrectinib and Ensartinib, have shown better results (60). For the above mentioned chemotherapeutic drugs and small molecule targeted therapy drugs, the Genomics of Drug Sensitivity in Cancer (GDSC) database was created. The immediate goal is to identify potential therapeutic biomarkers that may predict drug response (chemotherapeutic drugs, small molecule targeted drugs, and other drugs), while the ultimate goal is to improve the current status of cancer treatment based on biomarkers (44, 63). It has been shown that changes in the tumor genome directly affect the therapeutic effect of the tumor (64). With the emergence of novel compounds, the screening of predictive biomarkers in their early development process will have a profound impact on the entire process of new cancer drug development, including its design, development cost and final outcome (64). Based on the clinical and basic research background, researchers present the results of large-scale drug screening in human cancer cell lines in GDSC, a database that combines detailed genomic profiles and gene expression analysis to systematically provide biomarker identification patterns for drug sensitivity science for a variety of cancer drugs. In our study, we compared the IC50 levels of Osimertinib_1919, Sapitinib_1549, Acetalax_1804, Ibrutinib_1799, Erlotinib_1168, Gefitinib_1010, AZD3759_1915, Afatinib_1032, and Lapatinib_1558 between subgroups with high OS-score or low OS-score based on GDSC database. We found that LUAD patients with high OS-scores may be more sensitive to these nine chemotherapeutics drugs. Our study will provide reference for the treatment of LUAD.

Recent studies have shown that tumor microenvironment (TME) plays an important role in the development and treatment of tumors (65). TME refers to the microenvironment surrounding the occurrence, growth and metastasis of tumor cells, including not only the tumor cells themselves, but also the immune cells, inflammatory cells, fibroblasts, various signaling molecules, extracellular matrix and blood vessels (66). To fully understand and overcome the complexity of TME is helpful for clinicians to provide more feasible and precise individualized treatment plan for tumor treatment. The rapid development of single-cell sequencing, second-generation sequencing and other technologies has gradually deepened researchers’ understanding of the relationship between T cells and other immune cell populations and immunotherapy. Tumor-associated immune cells play an important role in tumor spread, recurrence, metastasis and influencing immunotherapy treatment (67). They can be used as biomarkers to predict the efficacy of immunotherapy drugs or predict the prognosis of patients (67). Increased levels of tumor-infiltrating lymphocytes (TILs), such as CD4+T cells and CD8+T cells, are associated with immunotherapy response and longer survival (68). Immune checkpoint inhibition activates existing TILs, which recognize and eliminate abnormal and tumor cells, and TILs play a key role in immunotherapy response. Studies have shown that increased T-cell infiltration and increased IFN-γ-related mRNA expression can increase ICIs (immune checkpoint inhibitors) benefit and significantly improve patient prognosis in a variety of tumor types (69, 70). In advanced NSCLC patients, increased expression of CD8+ TILs detected by IHC or CD8A mRNA transcripts was associated with prolonged PFS treatment with PD-L1 inhibitors, especially in combination with PD-L1 mRNA and protein expression, suggesting that integrated biomarkers may provide higher predictive value (71). Another study using multiple quantitative immunofluorescences to detect TIL in paraffin tumor specimens found that ICI treatment resulted in lasting clinical benefits and longer OS in NSCLC patients with increased CD3+ T-cell infiltration (72). In addition, studies have found that tumor-associated macrophages (TAMs) secrete interleukin-10 (interleukin-10), Il-10, Transforming growth factor-B (TGFb) and other immunosuppressive cytokines play a variety of tumor-promoting effects, which increase the density of TAM and inhibit other related immune cells (73).

The limitations this study remain. The OS-signature we constructed and validated by retrospectively using the public database hence, more prospective studies are needed for clinical practicability. We selected the three genes in the OS-signature to detect their expression in cell lines. Biological experiments in this study are lacking, and more wet experiments are needed to explore the function of related genes.

In conclusion, immunotherapy by regulating the immune microenvironment may become a promising new strategy for cancer treatment. The precise regulation of immune gene expression is the key to generate strong immunity and intervene the development of cancer. In our study, we found that patients with high OS-scores had lower immunomodulators levels except CD276, TNFSF9, and HMGB1. From a general view, the level of infiltrating immune cell populations decreased as the OS-scores increased. It is necessary to further study the tumor microenvironment (TME) of lung cancer.

## Data availability statement

The raw data supporting the conclusions of this article will be made available by the authors, without undue reservation.

## Author contributions

WW conceived, designed, and supervised the study. HP performed data analysis and drafted the manuscript. XL performed PCR experiments. YL and CW assisted with the analysis. All authors contributed to the article and approved the submitted version.
